# Cine DENSE MRI detects delayed mechanical activation of the left ventricular free wall in a canine model of heart failure with left bundle branch block

**DOI:** 10.1186/1532-429X-16-S1-O69

**Published:** 2014-01-16

**Authors:** Sophia Cui, Kenneth C Bilchick, Frederick H Epstein

**Affiliations:** 1 Biomedical Engineering, University of Virginia, Charlottesville, Virginia, USA; 2 Cardiovascular Medicine, University of Virginia, Charlottesville, Virginia, USA; 3 Radiology, University of Virginia, Charlottesville, Virginia, USA

## Background

Cardiac resynchronization therapy (CRT) benefits selected heart failure patients; however, CRT has a non-response rate of approximately 30-40%. Important factors that contribute to lead placement decisions and overall CRT success include determination of mechanical dyssynchrony, presence and location of scar, and the identification of late-activated segments. Recent imaging studies have reported progress in the first two areas. Previously, myocardial tagging showed promise for mapping mechanical activation time [1], but analysis of tagged images is time consuming. We investigated the use of cine DENSE to detect regions of late mechanical activation.

## Methods

**Figure 1:**
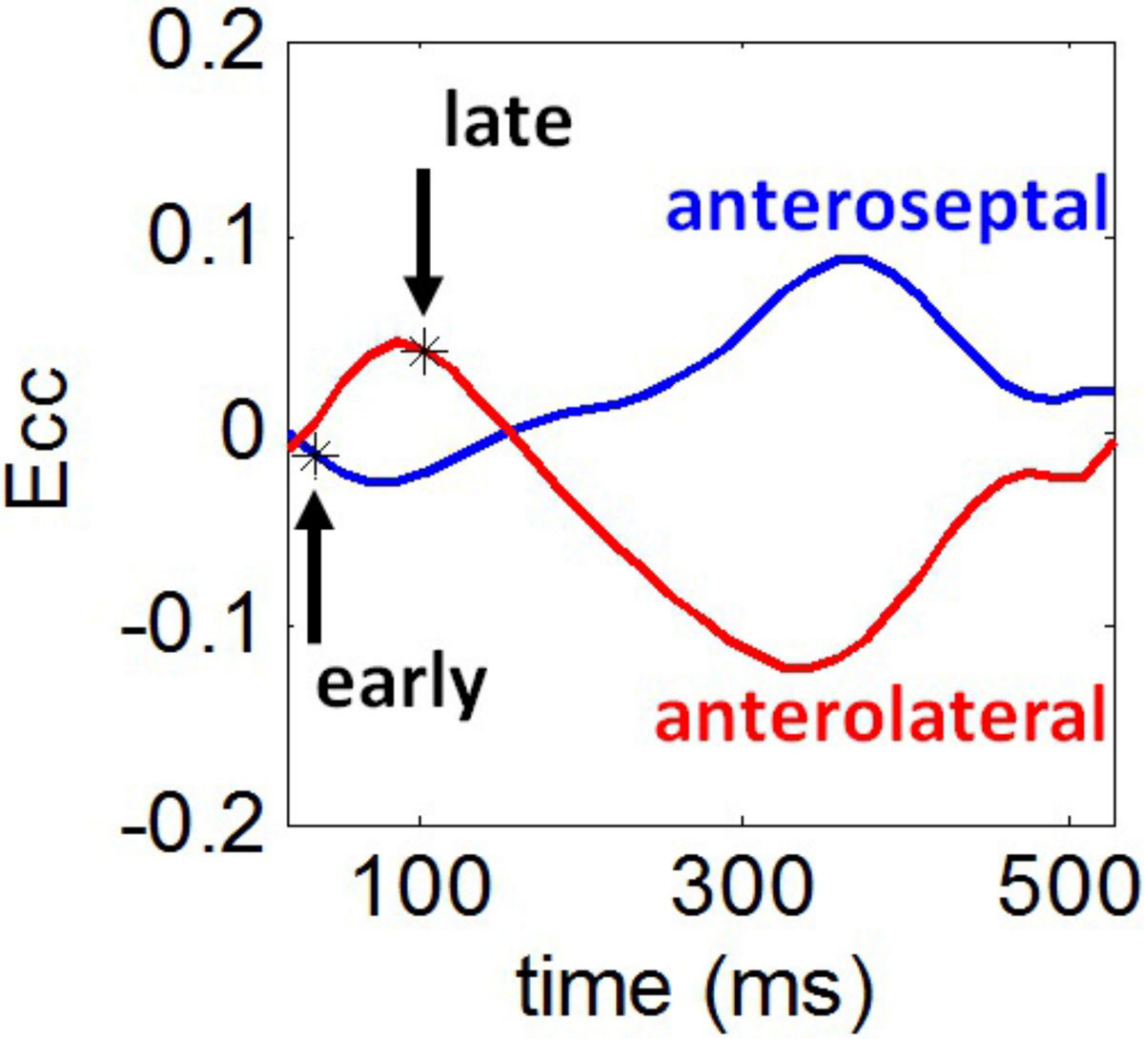
**Estimation of the time to mechanical activation**. Strain-time curves are shown for the anteroseptum (blue) and the anterolateral wall (red). The onset of contraction was defined as the first time where the slope of the strain curve is negative. The onset of contraction was early for the anteroseptum. In contrast, the anterolateral wall underwent early stretch, which was followed by a delayed onset of contraction. Regionally varying times of the onset of contraction were used to create mechanical activation time maps.

Cine DENSE at a temporal resolution of 17 ms, in-plane pixel size of 1.4 mm × 1.4 mm to 2.2 mm × 2.2 mm, and slice thickness of 8 mm was performed in standard short-axis planes in 4 canines with pacing-induced heart failure and left bundle branch block (LBBB) induced by catheter ablation. Using semi-automatic analysis methods [2,3], regional circumferential strain (Ecc) was determined from the mid-cavity short-axis DENSE images. The time to the onset of contraction (mechanical activation time) was defined as the time at which the slope of each Ecc curve first became negative, as shown in Figure 1. The mechanical activation time was computed for regions that were 5 × 5 pixels in size.

## Results

**Figure 2:**
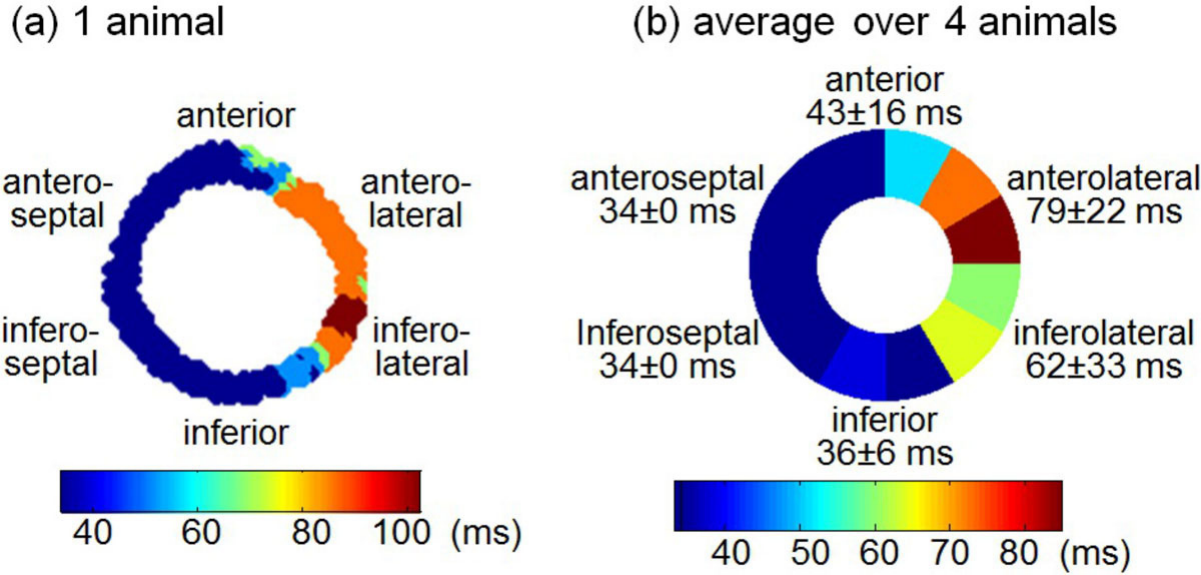
**(a) Pixel-wise mechanical activation time map of one canine with heart failure and LBBB**. (b) Segmental mechanical activation map of the average time to activation over all four animals. Color indicates the time at which the region was activated.

Cine DENSE showed that the septum underwent early activation, while the late-activated regions were consistently located in the anterolateral and inferolateral segments. These patterns were visualized using time-to-mechanical-activation maps, as shown in Figure 2a for one animal, and as shown in Figure 2b, which depicts the mean segmental values averaged for all 4 dogs. Early-activated segments underwent contraction within 34 ms of the imaging trigger, while the mean time for the latest activating segment was 79 ± 22 ms. Datasets from all 4 dogs depicted a temporal wavefront of activation beginning in the septum and spreading toward the left ventricular free wall. This mechanical pattern agrees with the electrical activation pattern previously reported in LBBB [4].

## Conclusions

Cine DENSE provides sufficient spatial and temporal resolution of LV strain to quantify the propagation of mechanical activation over the LV in a canine model of heart failure and LBBB. The time required to analyze cine DENSE data is much less than for conventional tagging, and completely automated methods have recently been described [5]. Cine DENSE shows promise for quantifying the extent of mechanical dyssynchrony [6], and identifying late-activated regions that would be good candidate sites for LV lead implantation.

## Funding

NIH K23 grant HL094761 and American Heart Association Grant-in-Aid 12GRNT12050301
